# Itchy Skin: A Challenging Differential Diagnosis Between Mycosis Fungoides and Sézary Syndrome

**DOI:** 10.7759/cureus.46427

**Published:** 2023-10-03

**Authors:** Cláudia Abreu Rocha, Margarida Drummond Borges, Guida Maria Santos, Miriam Sousa, Tânia Teixeira

**Affiliations:** 1 Family Medicine, Centro de Saúde de Machico, Serviço de Saúde da Região Autónoma da Madeira (SESARAM), Madeira Island, PRT; 2 Family Medicine, Centro de Saúde do Caniço, Serviço de Saúde da Região Autónoma da Madeira (SESARAM), Madeira Island, PRT; 3 Pathology, Hospital Central do Funchal, Madeira Island, PRT

**Keywords:** mycosis fungoidis, cutaneous t cell lymphoma, generalized rash, sézary syndrome, pruritus

## Abstract

Primary cutaneous lymphomas represent a diverse spectrum of T-cell and B-cell lymphomas with their primary skin manifestation. Among these, mycosis fungoides (MF) and Sézary syndrome (SS) represent classic forms of cutaneous T-cell lymphomas (CTCLs). This report details the case of a 67-year-old female who presented with longstanding pruritic skin lesions, initially misdiagnosed and managed as eczema. The diagnostic process ultimately revealed the presence of Sézary cells in the peripheral blood smear (PBS). The SS diagnosis was confirmed based on CD4 positivity and CD7 negativity as determined by flow cytometry. The disease was staged as IVA1 (T2N0M1B2). The patient exhibited partial improvement with oral corticosteroid therapy. This report underscores the critical importance of integrating clinical evaluation and blood findings to distinguish between MF and SS. The progression of a circulating clone signals a poor prognosis, requiring surveillance and consideration of targeted therapies to enhance patient outcomes and improve their quality of life. Early detection remains paramount in the management of these rare cutaneous lymphomas, which are associated with unique therapeutic challenges.

## Introduction

Primary cutaneous lymphomas encompass lymphomas that manifest in the skin, including a heterogeneous collection of cutaneous T-cell lymphomas (CTCLs) and cutaneous B-cell lymphomas (CBCLs). Within CTCLs, mycosis fungoides (MF) and Sézary syndrome (SS) represent classic forms [1].

Mycosis fungoides accounts for the majority of cases, initially manifesting in the skin with the potential for local or disseminated progression to the organs and lymph nodes. Its course varies and can progress to SS [1].

The SS is a rare subtype of cutaneous T-cell lymphoma, comprising 5% of cutaneous lymphomas. It is characterized by the presence of a malignant clone that primarily affects the skin and lymph nodes, as well as other organs and systems [2, 3]. It has an insidious progression that can manifest as a pruritic rash, progressing to erythroderma and the eventual appearance of adenopathy, lymphocytosis, hepatosplenomegaly, and B symptoms, including fever, night sweats, and weight loss [4].

This condition can be challenging to differentiate from other benign skin conditions. The diagnosis involves a thorough physical examination to analyze dermatological lesions, exclude other etiologies, assess the involvement of other systems, and determine the presence of adenopathy. The diagnostic confirmation is based on histological findings of skin lesions after lesion biopsy analysis. This syndrome presents neoplastic T cells, known as Sézary cells, in the peripheral blood with abnormal nuclei [5].

Treatment modalities encompass skin-targeted and systemic approaches involving radiotherapy, chemotherapy, corticosteroids, and phototherapy [6].

Unfavorable prognostic factors include extensive skin involvement (>80% of body surface), high tumor burden in the blood, extensive extracutaneous participation, and advanced age [7].

## Case presentation

**Figure 1:**
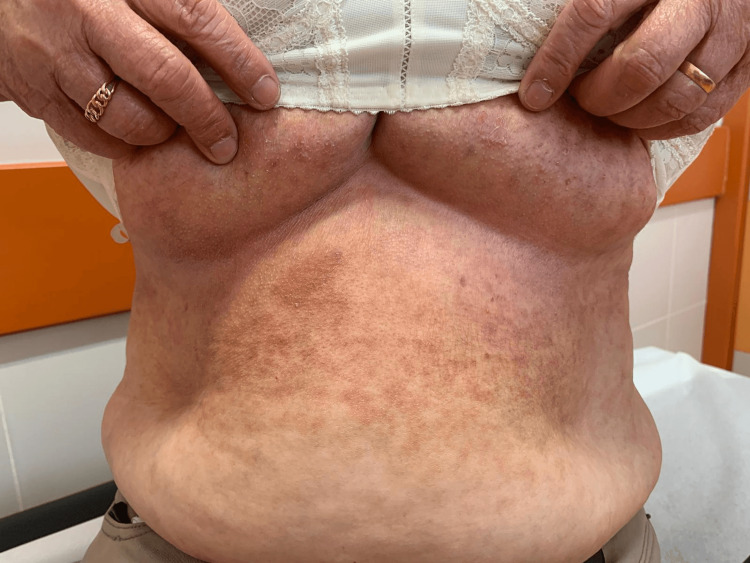
Skin lesions Macular and maculopapular skin lesions are scattered across the abdomen and in the inframammary region. We can observe scarring lesions secondary to itching.

**Figure 2:**
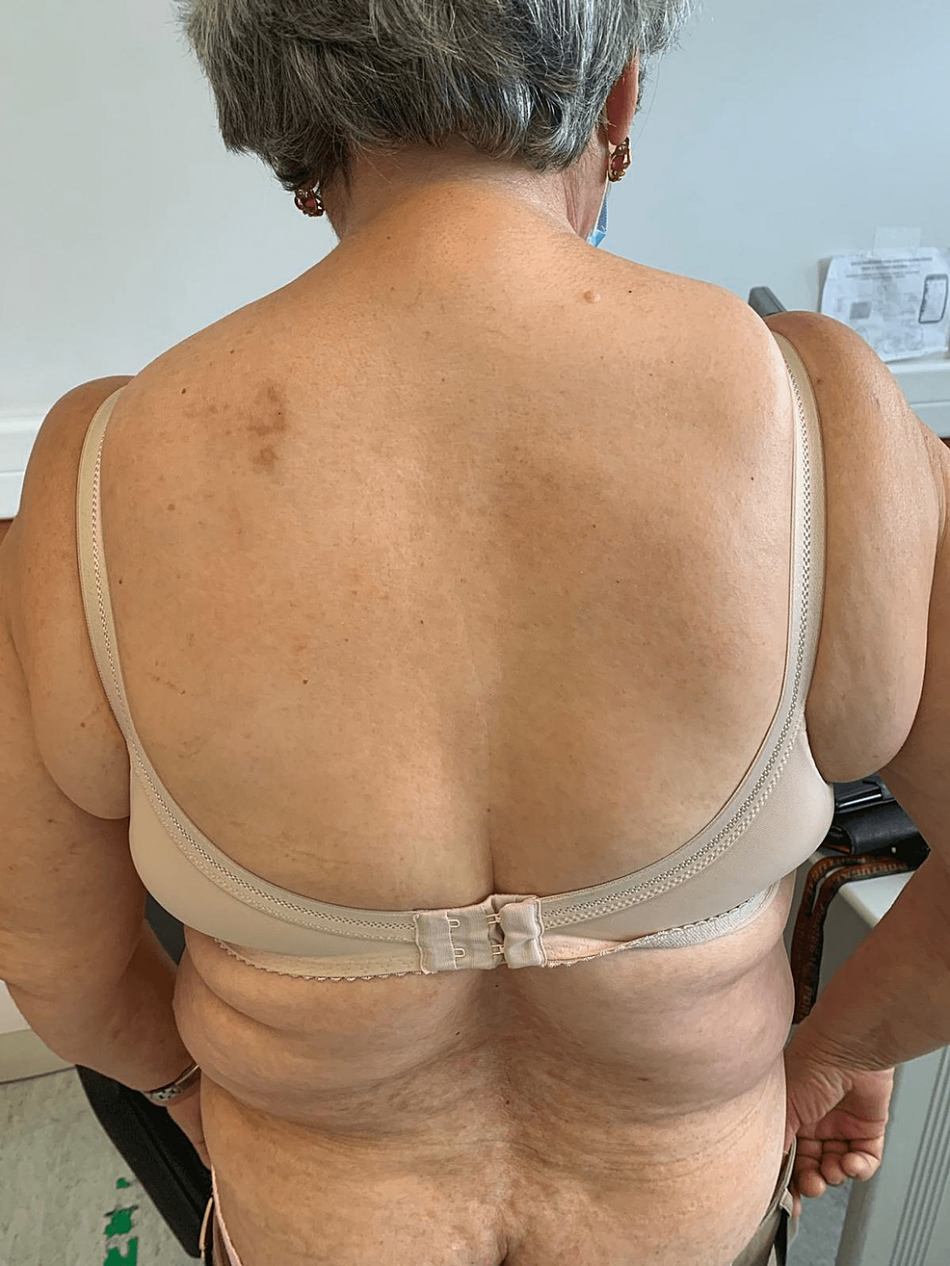
Skin lesions Macular and maculopapular skin lesions with an erythematous base spread across the trunk.

**Figure 3:**
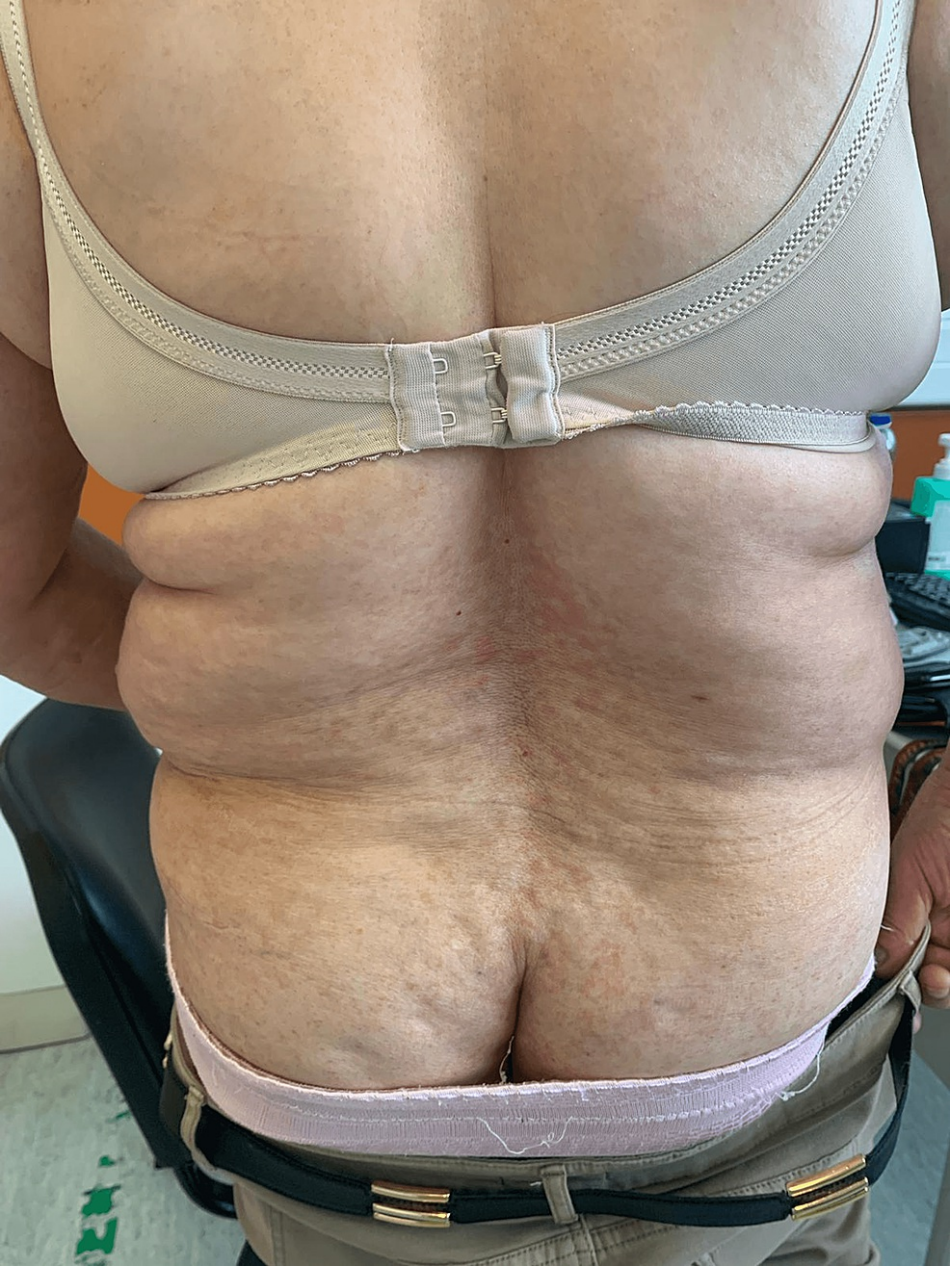
Skin lesions Macular and maculopapular skin lesions with an erythematous base spread across the trunk and back.

A 67-year-old female, a former cook with obesity and dyslipidemia, was chronically treated with simvastatin 40 mg. She consulted her general practitioner due to the recent exacerbation of skin lesions accompanied by severe generalized pruritus. She denied any weight loss, asthenia, night sweats, or other associated symptoms. On physical examination, the mucous membranes appeared hydrated and well-perfused, with macular lesions in the left scapular region and maculopapular lesions on the abdomen, trunk, and inframammary folds bilaterally (Figures 1-3).

There was no presence of alopecia or nail alterations. Additionally, there were no palpable peripheral lymphadenopathies or hepatosplenic alterations.

According to the patient, pruritic lesions manifested more than a decade ago, particularly in the inframammary folds. Initially, her physician prescribed topical antifungal treatment, suspecting dermatophytosis, but this approach yielded no improvement. Potential hypersensitivity reactions to amoxicillin were taken into consideration and subsequently investigated through immunoallergology, but no confirmation of allergies was found.

Due to the persisting immune-related complaints, the patient was referred to an external dermatology consultation. Initially, the suspicion of candidiasis led to the prescription of oral antifungal medication. However, due to no clinical improvement, the diagnosis was reassessed and altered to eczema. Treatment included hydroxyzine three times daily, oral prednisolone at a 20 mg dosage in short cycles, and the application of tacrolimus ointment. The symptomatology was only partially improved.

Considering the prolonged clinical course involving persistent skin lesions and pruritus, refractory to treatment, the physician explored rarer diagnostic hypotheses and requested an analytical evaluation.

**Figure 4:**
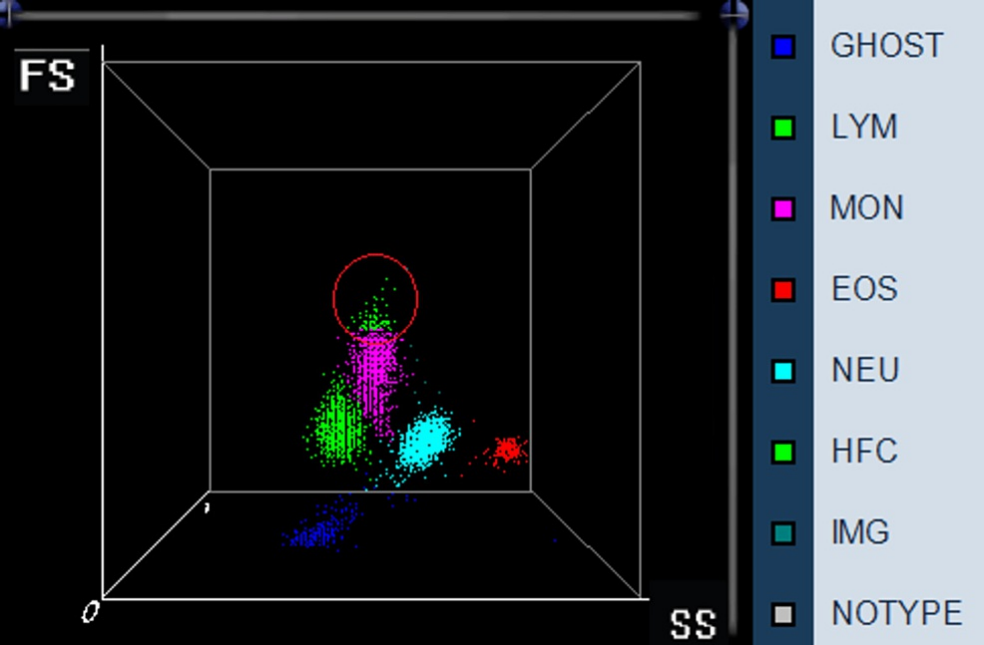
Blood count scatter plots X-axis, SS: side scatter; Y-axis, FS: forward scatter; tagged population with red circle: abnormal lymphocyte population

The sole deviation observed in the blood count was the presence of two distinct lymphocyte populations, as seen in the blood count scatter plots (Figure 4).

**Figure 5:**
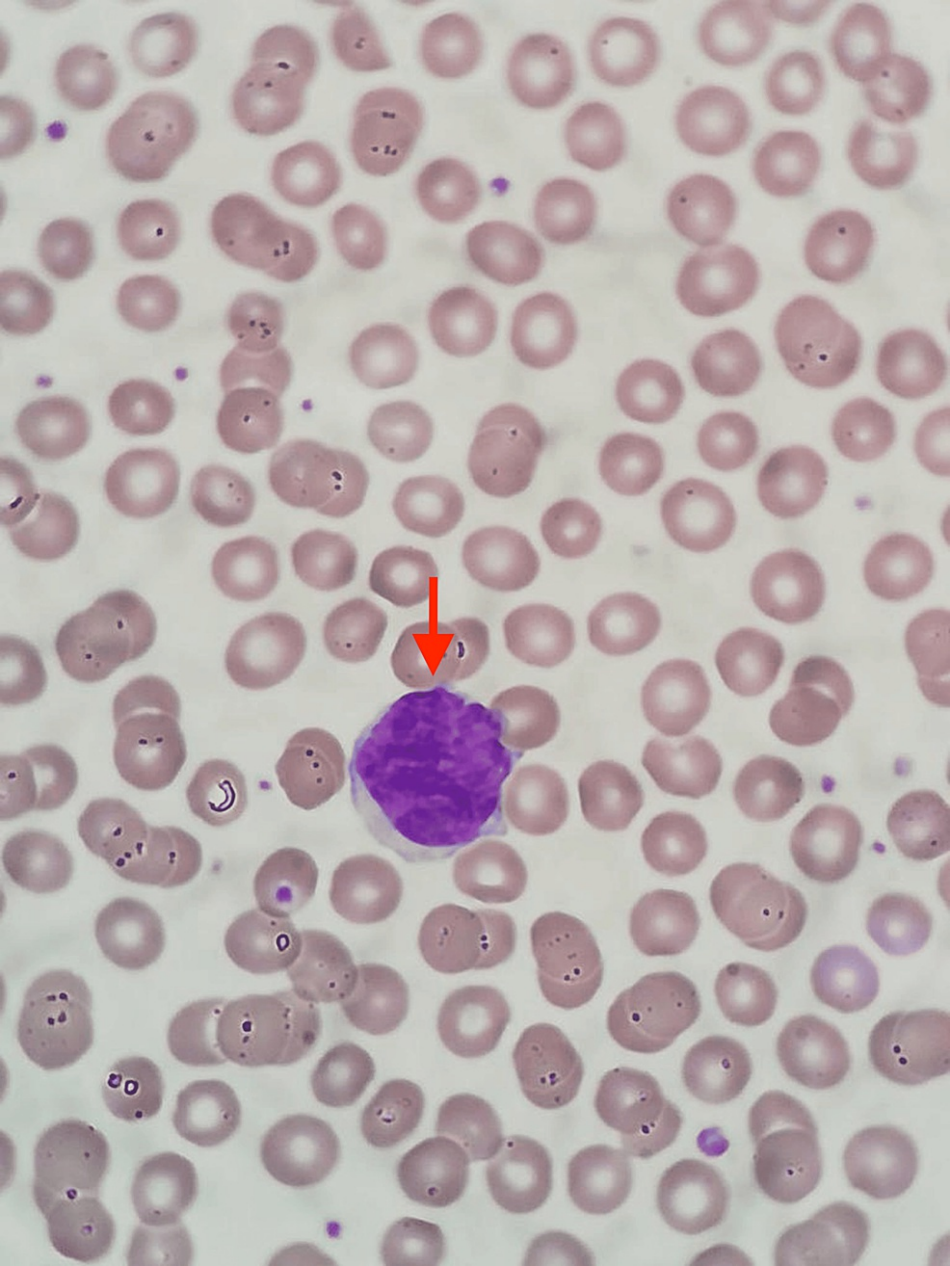
Peripheral blood smear; the red arrow shows one Sézary cell

**Figure 6:**
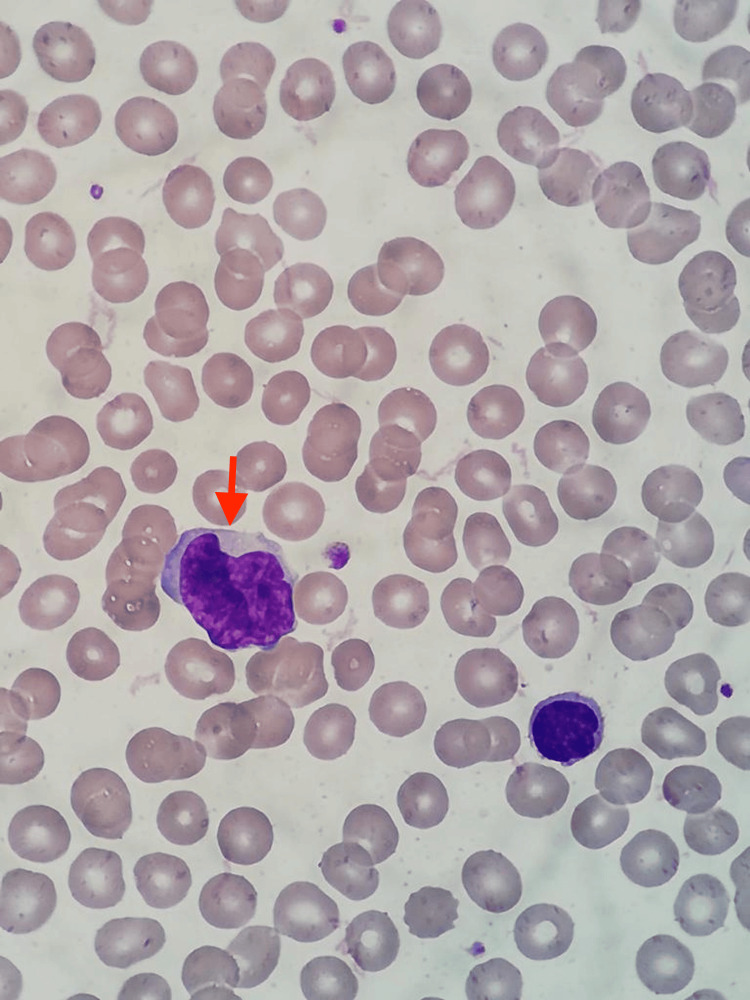
Peripheral blood smear; the red arrow shows one Sézary cell

Subsequently, a peripheral blood smear (PBS) was conducted, which revealed the presence of 1,100 atypical lymphocytes/μl (Table 1), characterized in the PBS by size two to three times larger than that of a small lymphocyte, a nucleus of mature chromatin, an anomalous pattern with a cerebriform appearance and scarce cytoplasm, being slightly basophilic, and compatibility with Sézary cells (Figures 5-6).

**Table 1:** The patient's complete blood count highlights the presence of 1,100 atypical lymphocytes/μl.

Parameter	Observed value	Unit	Reference range
Leukocytes	7.51	10^3/µl	4.2 – 10.8
Neutrophils	54	%	54 – 62
Lymphocytes	22	%	25 – 33
Monocytes	6	%	3 - 7
Eosinophils	2	%	1 - 3
Basophils	1	%	0 – 0.75
Atypical lymphocytes	15	%	
Neutrophils	4.1	10^3/µl	1.9 – 7.2
Lymphocytes	1.7	10^3/µl	1.2 – 3.4
Monocytes	0.5	10^3/µl	0.3 – 0.9
Eosinophils	0.2	10^3/µl	0.0 – 0.6
Basophils	0.1	10^3/µl	0.0 – 0.1
Atypical lymphocytes	1.1	10^3/µl	
Erythrocytes	4.36	10^6/µl	3.91 – 5.07
Hemoglobin	13.9	g/dL	11.9 – 14.9
Hematocrit	41	%	34 - 44
Platelets	247.0	10^3/µl	144 - 440

The patient was referred to a clinical hematology consultation. Flow cytometry of the peripheral blood lymphoid population was positive for CD4 and negative for CD7 (Table 2).

**Table 2:** Immunophenotyping by flow cytometry The results of immunophenotyping by flow cytometry of the peripheral blood lymphoid population, skin biopsy, and bone marrow aspiration

Immunophenotyping	Blood	Skin	Bone marrow aspiration
CD2	90%		92%
CD3	83%	Positive	90%
CD4	81%	Positive	88%
CD5	80%	Positive	Negative
CD7	Negative		Negative
CD8	2%	Negative	Negative
CD20	Negative	Negative	Negative
CD30		Negative	

A bone marrow biopsy, conducted at the posterior superior iliac crest, indicated a 6% infiltration of lymphoma cells, which were CD4+ and CD7-as determined by flow cytometry phenotyping. Computed tomography of the chest, abdomen, and pelvis did not show enlarged lymph nodes or visceral lesions. A biopsy of a skin lesion revealed an inflammatory infiltrate in the papillary dermis, consisting of small to intermediate, irregular mitotic lymphocytes, without significant epidermotropism. The atypical lymphocytes were CD3+, CD4+, CD8-, and CD20- (Table 2), confirming the diagnosis of cutaneous T-cell lymphoma.

The disease was staged as IVA1 (T2N0M1B2), currently presenting diagnostic criteria for Sézary syndrome. Following a multidisciplinary meeting, the decision was made to initiate treatment with oral prednisolone (5 mg/day) on a chronic basis and hydroxyzine as needed. The patient currently has an Eastern Cooperative Oncology Group (ECOG) performance status of level 0 and is able to carry out all activities. Currently, the patient exhibits an improvement in symptoms, although complete remission has not been achieved. Symptomatic surveillance remains crucial (at the skin, adenopathy, blood, and visceral levels), as well as the monitoring of potential complications associated with prolonged corticosteroid therapy and the consideration of an alternative therapeutic approach.

## Discussion

In this case report, the patient presented with pruritic erythematous patches scattered over more than 10% of the body surface, without plaques, tumors, or erythroderma. This complaint had persisted for an extended period, spanning over a decade, and exhibited a fluctuating pattern, suggesting an indolent disease course [3].

The refractoriness to therapy with oral corticosteroids in short cycles, topical immunosuppressants, and antihistamines raised suspicions regarding an alternative diagnosis for eczema [7,8]. This suspicion could have been addressed primarily with a skin biopsy of an affected area [9]. However, at the time of diagnosis, the disease had spread to the blood, with the presence of Sézary cells in the peripheral blood smear, resulting in a high tumor burden (over 1000 cells per µl) [10]. Furthermore, blood flow cytometry confirmed CD4 positivity and CD7 negativity, with a ratio exceeding 40%, ultimately allowing for the diagnosis of SS [11].

The SS is a rare and aggressive variant of CTCL, typically characterized by erythroderma and lymphadenopathy [12]. Therefore, an initial diagnosis of SS may not be entirely adequate, with the most plausible hypothesis being a long-standing MF that has progressed to a more advanced stage [3]. Blood involvement is an important negative factor, regardless of the patient's prognosis [13].

The patient's skin lesions improved with ongoing oral corticosteroid treatment. However, regular surveillance will be required regarding the possibility of skin and/or blood disease progression or adenopathic and/or visceral dissemination. Thus, considering treatments more specific to CTCL [14], such as monoclonal antibodies or conventional chemotherapy, might become imperative. Additionally, the use of these therapies may reduce the need for corticosteroids, often associated with adverse effects such as osteoporosis, cardiovascular disease, and metabolic disease, with long-term use.

## Conclusions

Cutaneous T-cell lymphomas can manifest in various cutaneous manifestations. Consequently, the integration between the clinical assessment and the blood analyses is crucial for the differential diagnosis between MF and SS. Circulating clone progression implies a poor prognosis, regardless of the diagnosis. Despite being an incurable disease, an early diagnosis enhances the patient’s quality of life and survival, which is why the physician should be aware of the rare causes of insidious complaints associated with therapeutic failure.
